# 

**Published:** 1866-03

**Authors:** 


### CHICAGO

### MEDICAL JOURNAL.

Vol. XXIII. MARCH, 1866. No. 3.

CHICAGO:

JAMES BARNET, PRINTER, 191 LAKE STREET.

